# Introduction

**DOI:** 10.1155/S1463924692000087

**Published:** 1992

**Authors:** Peter B. Stockwell


					Journal of Automatic Chemistry, Vol. 14, No. 2 (March-April 1992), p. 35
Special Issue: International Symposium on
Laboratory Automat .on and Robotics 1991.
Management Papers
Volume 14, Number 2, is devoted to papers presented at the ninth International Symposium on
Laboratory Automation and Robotics, which was held in Boston, USA from 20 to 23 October 1991.
The issue includes five papers on Managing Automation and Robotics, plus the conference's plenary
lectures by David B. Weinstein and Dennis S. France and Bernd Schade.
A full report on the meeting was published in Journal ofAutomatic Chemistry, Vol. 13, No. 6, together
with the abstracts of all the papers and posters presented at the Symposium.
The papers in this issue are reprinted, with permission, from the Proceedings ofthe 1991 International
Symposium on Laboratory Automation and Robotics. I would like to thank all those at the Zymark
Corporation, especially Sharon Correia, who have made this special issue possible.
Readers might wish to note the dates for the tenth International Symposium on Laboratory
Automation and Robotics: 25 to 28 October 1992. For details about papers, posters or registrations
readers should contact Sharon Correia, Zymark Corporation, Zymark Center, Hopkinton,
Massachusetts 01748, USA (Tel.: 508 435 9500; Fax: 508 435 3439).
CONTENTS
Managing robotics in the generic pharmaceutical arena, M. Schettter, p. 37.
The laboratory of the 1990s--Planning for total automation, L. A. Brunner, p. 43.
Strategic planning for bioanalytical automation: managing growth successfully, J. j. Tomlinson,
p. 47.
Selection criteria for laboratory robotic application personnel, P. W. Rulon, p. 51.
Measuring the effects of laboratory automation: The power of empirically derived models,
G. Fitzgerald and J. Swanson, p. 55.
Jumping into the 20th century before it is too late: is laboratory robotics still in its infancy?,
D. B. Weinstein and D. S. France, p. 59.
Quality control in the year 2000, B. Schade, p. 65.
PETER B. STOCKwELL
35

				

